# Photoprotection in a desert moss: dynamic excitation quenching during the hydration cycle of the *Syntrichia caninervis*

**DOI:** 10.1007/s11120-026-01230-4

**Published:** 2026-07-10

**Authors:** Simona Streckaite, Jevgenij Chmeliov, Vilius Čirgelis, Marius Franckevičius, Lena Golubewa, Benfeng Yin, Danielis Rutkauskas, Chunhong Yang, Leonas Valkunas, Yuanming Zhang, Bruno Robert

**Affiliations:** 1 Department of Molecular Compound Physics, Center for Physical Sciences and Technology, Saulėtekio Ave. 3, Vilnius, 10257 Lithuania; 2 Institute of Chemical Physics, Faculty of Physics, Vilnius University, Saulėtekio Ave. 9, Vilnius, 10222 Lithuania; 3 State Key Laboratory of Ecological Safety and Sustainable Development in Arid Lands, Xinjiang Institute of Ecology and Geography, Chinese Academy of Sciences, Urumqi, Xinjiang 830011 China; 4 Key Laboratory of Vegetation and Environmental Change, Institute of Botany, Chinese Academy of Sciences, Beijing, 100093 China; 5 Institute for Integrative Biology of the Cell, Université Paris-Saclay, CEA, CNRS, Gif-sur-Yvette, 91198 France

**Keywords:** Syntrichia caninervis, Photosynthesis, Photosystems, Streak camera, Fluorescence

## Abstract

Desiccation-tolerant mosses survive extreme water loss by activating efficient photoprotective mechanisms that prevent damage to the photosynthetic apparatus. The most resistant moss *Syntrichia caninervis* (*S. caninervis*) represents an important model for studying the molecular basis of dehydration resilience; however, the membrane and protein level processes, which enable rapid recovery of photosynthetic activity upon rehydration, remain insufficiently understood. In this work, we investigate the fluorescence (FL) dynamics of the photosynthetic apparatus of *S. caninervis* under three hydration states (hydrated, dehydrated and desiccated) in whole leaves using time-resolved FL spectroscopy. Time-resolved measurements with ~ 10 ps resolution were performed to resolve excitation dynamics in both photosystems I (PSI) and II (PSII). The complex FL datasets were analysed using the spectral decomposition approach. Dehydrated and desiccated samples exhibit substantially reduced FL intensity compared to hydrated moss in steady-state experiments, indicating efficient quenching of chlorophyll excitation upon drying. The time-resolved FL measurements revealed that excitation transfer rates and the nature of quenched states strongly depend on hydration level in *S. caninervis*. In the hydrated state, FL dynamics are consistent with functional photosystems in plants. Upon dehydration, non-photochemical quenching is activated in both PSII and PSI, with PSII quenching persisting even at low temperatures. We therefore conclude, that *S. caninervis* employs few hydration-dependent photoprotective regimes, including distinct quenching mechanisms at both photosystems. Our results suggest the presence of ultrafast quenching processes under severe dehydration, which are progressively relieved during rehydration and replaced by alternative mechanisms supporting the recovery of photosynthetic activity.

**Supplementary Information:**

The online version contains supplementary material available at 10.1007/s11120-026-01230-4.

## Introduction

Global climate change and the associated increase in aridity pose major challenges for terrestrial vegetation. Understanding the survival strategies of plants capable of withstanding extreme environments has therefore become an important topic in plant biology. Drought tolerance has been intensively studied since the 1970s (see the review on early works (Oliver et al. 2005) and remains a highly relevant research area today (Arslan et al. 2025; Cardoso Neto et al. 2025; Kawamura et al. 2026), particularly because of its significance for agriculture. Bryophytes (*Bryophytae*), the mosses, have attracted particular attention, as this division includes numerous species exhibiting varying degrees of drought tolerance (Morales-Sánchez et al. 2022; Ndhlovu et al. 2025; Singh et al. 2025; Yang et al. 2025). Among them, the desert moss *Syntrichia caninervis* (*S. caninervis*) is one of the most stress-resistant bryophytes, thriving under conditions of intense solar radiation, limited precipitation, prolonged snow cover, and temperature fluctuations ranging from around − 50 °C to + 50 °C. It displays a remarkable tolerance to dehydration and desiccation (Li et al. 2024), as well as the ability to survive in both cold (Yin and Zhang 2016) and hot (Zhuo et al. 2025) environments. Its extraordinary resistance to harsh environmental conditions and radiations has even made this moss a candidate for astrobiological studies focused on extraterrestrial colonization (Li et al. 2024). *S. caninervis*, one of the dominant species of biological soil crusts in the Gurbantunggut Desert in northwest China, thus represents an invaluable model for addressing the molecular mechanisms underlying resilience in photosynthetic organisms.

*S. caninervis* possesses a specialized and highly efficient system for water collection and transport (Pan et al. 2016) and survives dehydration by entering a dormant state, in which metabolic and biochemical processes are largely suspended (Yang et al. 2023, 2025). Despite this metabolic inactivity, the moss retains a remarkable capacity to rapidly resume growth and photosynthetic activity within seconds to minutes after rehydration (Li et al. 2010; Proctor and Smirnoff 2000; Wu et al. 2012). The morphological and physiological traits underlying the exceptional resilience of *S. caninervis* have been extensively investigated at the cellular and organismal levels (Li et al. 2024; Pan et al. 2016; Wu et al. 2012; Zheng et al. 2011; Zhuo et al. 2025). However, its specialized photosynthetic machinery, particularly the mechanisms underlying the recovery of photosystem I (PSI) and photosystem II (PSII) activity in response to hydration state, remains poorly understood. The molecular and membrane-level mechanisms enabling the immediate recovery of photosynthetic activity upon rehydration, including the reorganization of pigment–protein complexes and energy transfer pathways during the dehydration–rehydration cycle, are particularly puzzling. It was determined that *S. caninervis* rapidly restores a fraction of PSII electron transport, excitation energy transfer, oxygen evolution, and charge separation within minutes of rehydration (Li et al. 2010; Proctor and Smirnoff 2000; Wu et al. 2012). This rapid recovery occurs in at least two phases: an initial fast phase (less than 1 min), during which approximately half of the activity of PSII (and probably PSI) is restored, followed by a slower phase (~ 5 min) leading to the full restoration of the photosynthetic activity of the moss. These phases were shown to occur without the need for *de novo* chloroplast protein synthesis (Li et al. 2010; Proctor and Smirnoff 2000) and were proposed to involve *de novo* chlorophyll synthesis (supported by pre-existing enzymatic machinery) and a rapid structural reorganization of PSII. In the moss *Bryum argenteum*, the amount of both chlorophyll pigments and photosynthetic proteins was observed to decrease upon drying, and a fast recovery of the about 50% of the photosynthetic apparatus was detected after 5 min of rehydration in the light (Li et al. 2014). The molecular mechanisms underlying these astonishingly fast processes remain to be fully understood.

In this work, we investigate the fluorescence (FL) dynamics of the photosynthetic apparatus in the moss *S. caninervis* across different hydration levels using a combination of steady-state and time-resolved FL spectroscopy. Most studies to date have relied on steady-state FL measurements. However, in steady-state measurements, the detected signal intensity depends on both the amount of absorbed excitation light and the fluorescence quantum yield, which is proportional to the fluorescence lifetime, of the emitting species. As a result, strongly quenched components contribute little, if at all, to steady-state FL spectra, which is the reason why PSI is barely detectable in room-temperature (RT) FL spectra. To overcome these limitations, we performed streak-camera-based time-resolved FL measurements to characterize whole leaves of *S. caninervis* in three hydration states: *hydrated*, *dehydrated*, and *desiccated*. The resulting complex FL patterns were analysed using a well-established spectral decomposition approach, which has proven successful in interpreting fluorescence from isolated light-harvesting complexes (LHCs) of diatoms and plants (Gelzinis et al. 2024; Mascoli et al. 2020), as well as from *Arabidopsis thaliana* wild type and mutants (Chmeliov et al. 2019). Our results demonstrate that excitation transfer rates and the nature of quenched states vary with the hydration level of *S. caninervis*, strongly influencing photosynthetic activity. We show that this drought-resistant moss exhibits different photoprotective mechanisms upon drying than those described in higher plants, involving ultrafast quenching, which enables efficient protection of the photosynthetic apparatus during extreme water loss and rapid recovery upon rehydration. We further speculate that dehydration-induced structural rearrangements may contribute to this photoprotective response.

## Materials and methods

### Sample preparation

*S. caninervis* moss was obtained from Gurbantunggut Desert in northwest China. A small clump of *S. caninervis* was placed in a Petri dish and stored in a closed laboratory drawer. The storage conditions were ambient temperature (20 °C) and a relative humidity of 20%, with no exposure to light. No water was supplied during storage (around 12 months). Moss samples in different hydration conditions were all prepared at a temperature of 23 °C, a relative humidity of 20%, and an illumination of approximately 20 µmol photons m^–2^s^–1^, after having been gently blown with a mild nitrogen flow to remove surface contaminants such as dirt, sand, or dust. All samples were measured in demountable circular quartz cuvettes with an optical path length of 0.01 mm (QS 124; Helma), in which leaves were slightly pressed between windows, and sealed with glue. Before measurements, samples were kept in the dark for at least 1 h.

*Dehydrated* samples consisted of small pieces of *S. caninervis* taken directly from their long-term storage conditions. *Hydrated* samples were obtained by placing the moss on a piece of filter paper soaked in deionized water. Several drops of deionized water were then added directly onto the plant material to ensure complete coverage with water. The samples were rehydrated under these conditions for 30 min, after which they were gently blown with a mild nitrogen flow to remove excess surface water and then sealed in the quartz cell. *Desiccated* samples were obtained by placing a small piece of dehydrated *S. caninervis* in a glass vial without lid and maintaining it for 6 h at 40 °C in an oven. Then, the sample was cooled down to RT and sealed into the circular quartz cell. After measurements, which involve cooling the samples down to 16 K, these desiccated samples were rehydrated following the same protocol as for preparing hydrated samples and re-measured to ensure the reversibility of the desiccation treatment. Such samples are labeled below as *Rehydrated*.

### Equipment

Picosecond time-resolved FL measurements were performed with a streak camera system (Hamamatsu C5680) using synchroscan regime. Samples were excited with a femtosecond Yb: KGW laser (Light Conversion Ltd.) generating 80 fs duration pulses at 1030 nm wavelength, which was halved to 515 nm using HIRO harmonics generator (Light Conversion Ltd.), at a repetition rate of 76 MHz. Excitation energy density was attenuated using neutral density filters to about few hundreds nJ cm^− 2^. Excitation was focused using fused silica plano-convex lens (LA4924; Thorlabs) with a focal length of 18 cm into ~ 100 μm spot on the sample. The time resolution of the system was ∼10 ps. Signal acquisition time was 20–30 min for each measurement. Although carotenoids absorb significantly at 515 nm, chlorophyll absorption also remains non-negligible at this wavelength, allowing efficient excitation of the photosynthetic apparatus. Since excitation energy absorbed by carotenoids is rapidly transferred to chlorophylls within the LHCs, the FL detected in our experiments predominantly originates from chlorophyll emission.

Additional time-resolved FL microscopy measurements were performed using a configuration different from that used for the spectroscopic time-resolved FL measurements. The microscopy measurements employed the same laser source and detection system, but the sample was placed in a microscope equipped with a 10× plan achromatic objective (0.25 NA, 10.6 mm WD; Olympus) rather than before the elliptical mirrors which collect FL for the monochromator in the spectroscopic streak-camera setup. Consequently, the excitation spot size was ~ 15 μm for the microscopy measurements. Microscopy measurements were performed to assess the spatial heterogeneity of the samples at different hydration states.

Pico-to-nanosecond time-resolved FL measurements were performed with F920 spectrometer (Edinburgh Instruments). Fluorescence decay kinetics were recorded using time-correlated single photon counting (TCSPC). The excitation source was a picosecond-pulsed diode laser EPL-470 (Edinburgh Instruments) emitting ∼72 ps pulses at a repetition rate of 5 MHz.

Temperature dependence measurements were performed cooling the sealed quartz cuvettes from RT down to 16 K using a cold finger in a closed helium cycle cryostat (Janis CCS-100/204).

### Data treatment

Time-resolved FL measurements performed with the streak-camera system exhibited complex decay kinetics that varied across the emission spectrum. After background subtraction, the experimental kinetics at selected wavelength intervals were fitted using single-, double- or triple-exponential decay functions $\:\left({\sum\:}_{n}{A}_{n}\mathrm{exp}\left(-t/{\tau\:}_{n}\right)\right)$ convoluted with a Gaussian-shaped pulse representing the instrument response function (IRF; full width at half maximum, FWHM = 10 ps). The obtained lifetimes and their relative amplitudes are summarized in Table 1. The amplitude-averaged mean lifetime was defined as $\:{\tau\:}_{\mathrm{a}\mathrm{v}}={\sum\:}_{n}{A}_{n}{\tau\:}_{n}/{\sum\:}_{n}{A}_{n}$.

To extract the dominant spectral components, present in the collected data, the raw time-resolved fluorescence spectra $\:{F}_{\mathrm{r}\mathrm{a}\mathrm{w}}\left(\lambda\:,t\right)$ were further analyzed in the following way. First, the contribution of the emission in the yellow–orange spectral region was removed. The emission in the 560–630 nm range was reasonably-well fitted (in the frequency domain) with a Gaussian lineshape, while the FL kinetics, $\:{K}_{0}\left(t\right)$, were found to be wavelength-independent across this region. After extrapolating this spectral lineshape $\:{S}_{0}\left(\lambda\:\right)$ to the longer wavelengths, its contribution was subtracted from $\:{F}_{\mathrm{r}\mathrm{a}\mathrm{w}}\left(\lambda\:,t\right)$ to yield a cleaner time-resolved fluorescence data $\:F\left(\lambda\:,t\right)$ in the PSI/PSII fluorescence region:

1 $$\:F\left(\lambda\:,t\right)={F}_{\mathrm{r}\mathrm{a}\mathrm{w}}\left(\lambda\:,t\right)-{S}_{0}\left(\lambda\:\right)\cdot\:{K}_{0}\left(t\right),$$

as illustrated in *Supplementary Material*, Fig. S6. The resulting $\:F\left(\lambda\:,t\right)$ dataset was then further analyzed using multivariate curve resolution (Lawton and Sylvestre 1971)—a method that has been successfully applied to study temperature-dependent absorption(Chmeliov et al. 2013; Trinkunas et al. 2012) and time-resolved fluorescence spectra(Chmeliov et al. 2016, 2019; Farooq et al. 2018; Gelzinis et al. 2024; Mascoli et al. 2020) of various photosynthetic complexes. The $\:F\left(\lambda\:,t\right)$ data was decomposed into two dominating spectral components:

2 $$\:F\left(\lambda\:,t\right)\approx\:{S}_{1}\left(\lambda\:\right)\cdot\:{K}_{1}\left(t\right)+{S}_{2}\left(\lambda\:\right)\cdot\:{K}_{2}\left(t\right),$$

where $\:{S}_{1}\left(\lambda\:\right)$ and $\:{K}_{1}\left(t\right)$ correspond to the steady-state spectrum and kinetics of the ~ 685 nm PSII-dominated emission, and $\:{S}_{2}\left(\lambda\:\right)$ and $\:{K}_{2}\left(t\right)$ account for the red-shifted spectral component. At RT, $\:{S}_{2}\left(\lambda\:\right)$ can be attributed primarily to PSI; but at lower temperatures it might also contain the FL signal originating in the red-emitting states of the PSII antenna complexes (Chmeliov et al. 2016).

In a matrix form, Eq. 2 can be written as $\:\mathbf{F}=\mathbf{S}\cdot\:\mathbf{K},$ where $\:\mathbf{F}$ is an $\:n\times\:m$ matrix of the initial dataset taken at * n* wavelengths and * m* time points, $\:\mathbf{S}$ is an $\:n\times\:2$ matrix containing the steady-state FL spectra of both components, and $\:\mathbf{K}$ is a $\:2\times\:m$ matrix containing their corresponding kinetics. Matrix factorization was performed using the multiplicative update algorithm (Berry et al. 2007), the optimizations were carried out for 200 times starting from random non-negative initial matrices **S** and **K**. To reduce the intrinsic ambiguity of such a decomposition, in addition to the typical non-negativity constraints on both matrices **S** and **K**, we performed a simultaneous fit for the three RT datasets obtained for the hydrated, dehydrated and desiccated samples, while requiring the matrix **S** to be identical across all three hydration states. The same procedure was applied to the dataset measured at other temperatures. The quality of such decomposition was validated by reconstructing FL kinetics at different wavelengths and FL spectra at different time delays. Comparison with the corresponding slices from $\:F\left(\lambda\:,t\right)$ indicated that the obtained two components indeed capture the major characteristics of the time-resolved FL spectra.

## Results

**Fig. 1:**
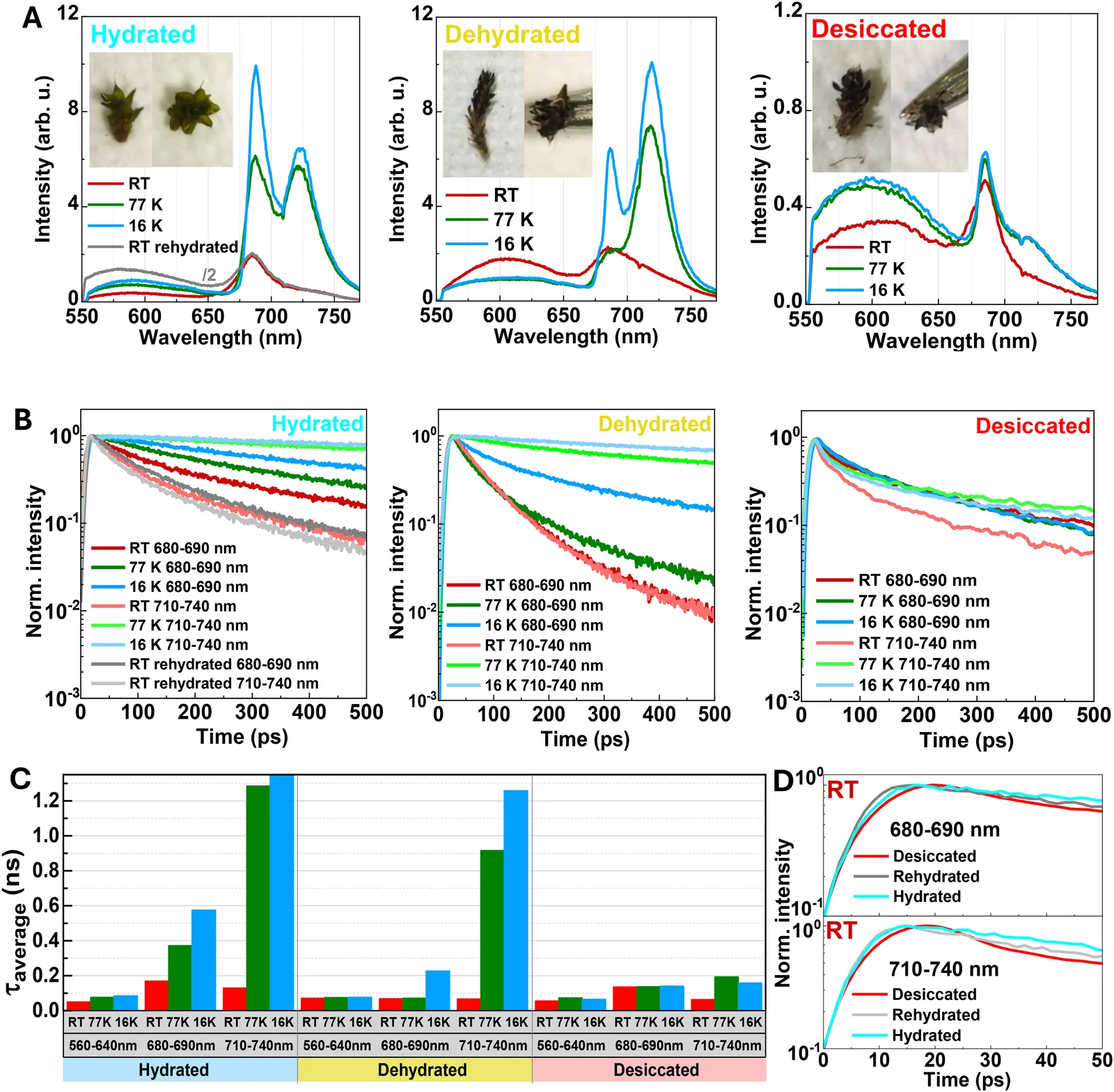
Hydrated, dehydrated and desiccated *S. caninervis* at RT, 77 K and 16 K. **A**: 800-ps integrated FL spectra, **B**: normalized FL decay kinetics of 680–690 nm and 710–740 nm regions, **C**: average FL lifetimes of 3 spectral regions of interest, **D**: initial 50 ps of FL kinetics of desiccated sample, the same sample after rehydration, and hydrated sample. In **A** and **B**, the RT FL spectrum and kinetics of the desiccated sample that was first cooled down to 16 K, then heated back to RT, and then rehydrated (gray lines) is shown in comparison to hydrated sample. Measurement window—800 ps; laser repetition rate—80 MHz;$\:\:{\lambda\:}_{\mathrm{e}\mathrm{x}\mathrm{c}}=\:$515 nm. All spectra are normalized to the measurement time

FL dynamics experiments of *S. caninervis* samples in the three hydration states were performed within an 800-ps time window at different temperatures in order to capture not only PSII FL dynamics but also the much faster PSI component (Fig. 1). All measurements were performed on dark-adapted samples under low excitation fluences (few hundred nJ cm^− 2^). To verify whether the PSII reaction centers remain open or are closed under such conditions, additional measurements over several-ns time windows were also carried out (see *Supplementary Material*, Fig. S4) to capture the whole FL dynamics and verify long FL components of the samples. In the hydrated samples, a large (~ 50%) amplitude of the longest ~ 3.5 ns lifetime component (see *Supplementary Material*, Table S1) suggests the presence of the closed RCs. Meanwhile, in the dehydrated samples the longest lifetime component is shortened down to ~ 1–1.5 ns, with an amplitude of just ~ 1%, which indicates that either the absolute majority of the RCs are open or strong non-photochemical quenchers are present in the antenna.

Additional RT time-resolved FL measurements with streak camera spectroscopy system coupled with microscope were performed to check sample heterogeneity. It must be noted that, while time-resolved FL microscopy measurements at RTof hydrated samples yielded very similar FL spectra and decay kinetics across different spots of the samples (~ 15 μm in diameter), dehydrated moss samples showed greater variability in both spectral features and decay dynamics across different sample locations, indicating slightly increased local FL heterogeneity (see *Supplementary Material*, Fig. S5).

**Fig. 2:**
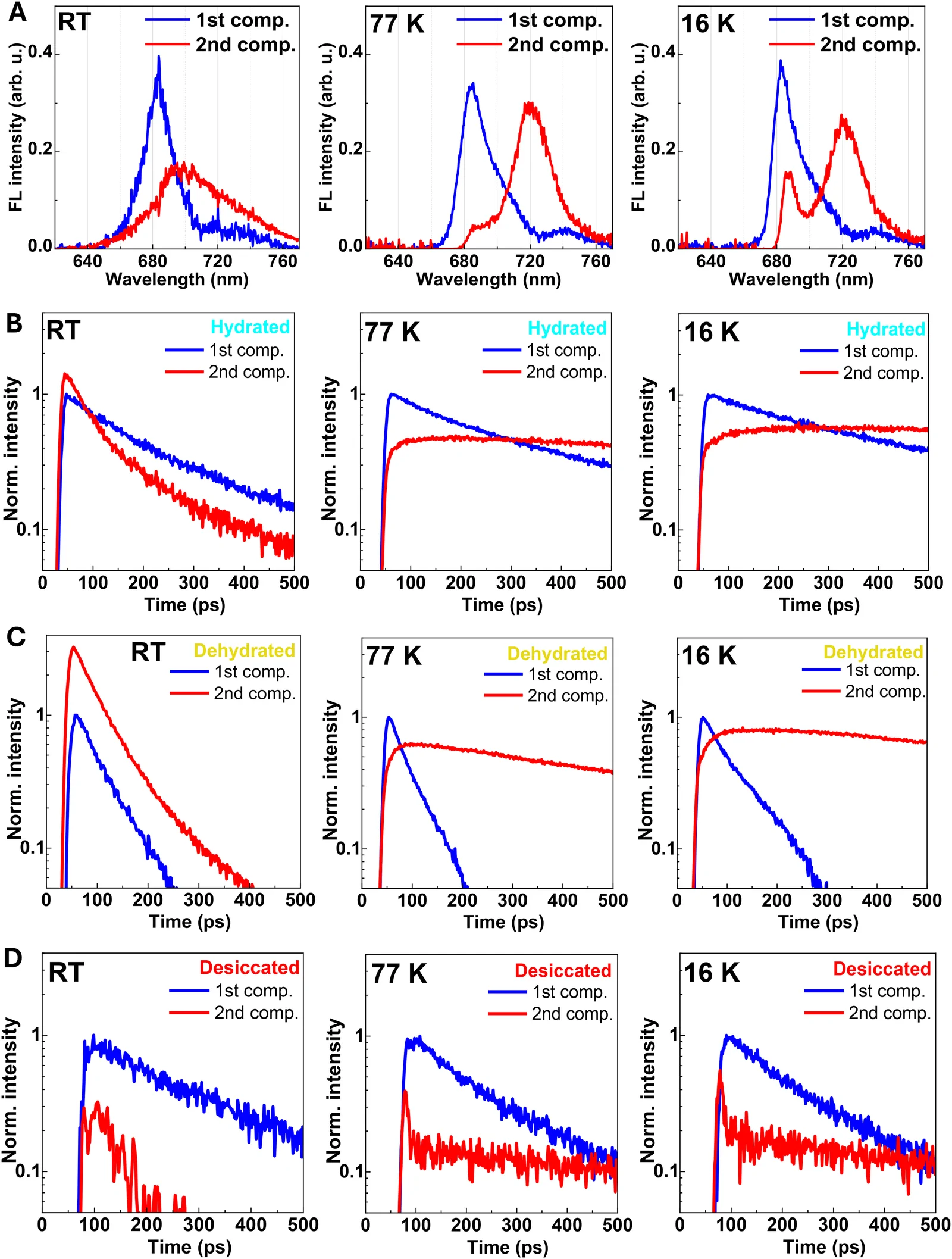
Spectral decomposition of hydrated, dehydrated and desiccated *S. caninervis* samples at RT, 77 K and 16 K. **A**: Steady-state FL spectra (normalized to the area) of the two spectral components corresponding to PSI and PSII regions, obtained by assuming them to remain the same across all three hydration states. **B–D**: FL kinetics corresponding to the components of spectral decompositions in **A**. The orange region was excluded from all spectral decompositions

The data were analyzed by spectral decomposition assuming that, at each temperature, the emission spectra in the PSI and PSII regions comprise the same spectral components in every hydration state of the moss (Fig. 2, see *Materials and methods* for details, and for the whole time window see Fig. S7). Those can indeed be described as the sum of the same two components at each temperature. This approach enables direct comparison of the hydration dependence of the spectral components extracted from the time-resolved FL data.

### Hydrated moss

At room temperature, the emission spectrum of hydrated moss comprises a main peak at 685 nm (Fig. 1A), consistent with typical fluorescence spectra of higher plants and green algae (Govindjee 1995), accompanied by a red shoulder at about 720 nm, which is blue–shifted relative to the typical 730–740 nm emission side–band. Upon cooling, the main emission band undergoes a red shift of approximately 2–3 nm and increases in intensity by several–fold. A prominent band at 723 nm appears at 77 K and below, a feature attributed to PSI emission (Govindjee 1995; Lamb et al. 2018; Strasser and Butler 1977). At these cryogenic temperatures, the 680–690 nm region decays faster than the 710–740 nm region, while the opposite is observed at room temperature. In the 710–740 nm region, the decays at 77 K and below are very similar, while FL decay slows down as temperature decreases in the 680–690 nm PSII–dominated region.

At room temperature, both spectral regions are best described by bi–exponential kinetics with a fast (around 60 ps) and a slower (around 320 ps) decay lifetime. Below 77 K, FL decay in the 680–690 nm region remains biexponential, whereas a single–exponential, slower, decay is observed for the 710–740 nm region. The mean lifetimes are compared in Fig. 1C, and detailed fitting parameters are provided in Table 1. Overall, the behavior of FL kinetics in both spectral regions, which accounts for the different excitation dynamics following photon absorption, are similar to that observed in *Arabidopsis thaliana* (Chmeliov et al. 2019; Wientjes et al. 2017).

In addition to these FL bands, a weak and broad emission is detected in the orange spectral region. Its intensity slightly increases as the temperature decreases, although small displacement in the measurement spot upon cooling may contribute to this effect. This band exhibits three–exponential FL decay kinetics that remain very similar at all temperatures (see Table 1 and *Supplementary Material*, Fig. S2).

**Table 1:** Amplitude–averaged FL lifetimes and FL lifetimes with their amplitudes in brackets of each component of multiexponential decay fits of FL kinetics of 560–640 nm, 680–690 nm and 710–740 nm spectral regions of interest of hydrated, dehydrated and desiccated *S. caninervis* measured at different temperatures.$\:{\lambda\:}_{exc}=\:$515 nm

	Region	τ_av_ [ns]	τ_1_ [ns] (A_1_ [%])	τ_2_ [ns] (A_2_ [%])	τ_3_ [ns] (A_3_ [%])
**Hydrated**					
RT	560–640 nm	0.050	0.01 (66)	0.061 (26)	0.349 (8)
	680–690 nm	0.169	0.058 (58)	0.319 (42)	-
	710–740 nm	0.129	0.056 (73)	0.326 (27)	-
77 K	560–640 nm	0.076	0.014 (59)	0.073 (31)	0.450 (10)
	680–690 nm	0.372	0.150 (58)	0.680 (42)	-
	710–740 nm	1.285	1.285 (100)	-	-
16 K	560–640 nm	0.085	0.015 (57)	0.077 (32)	0.451 (11)
	680–690 nm	0.575	0.211 (57)	1.055 (43)	-
	710–740 nm	1.890	1.890 (100)	-	-
**Dehydrated**					
RT	560–640 nm	0.071	0.016 (62)	0.081 (29)	0.410 (9)
	680–690 nm	0.068	0.056 (94)	0.248 (6)	-
	710–740 nm	0.067	0.055 (94)	0.262 (6)	-
77 K	560–640 nm	0.075	0.013 (57)	0.070 (32)	0.406 (11)
	680–690 nm	0.071	0.027 (59)	0.090 (34)	0.367 (7)
	710–740 nm	0.916	0.167 (33)	1.275 (67)	-
16 K	560–640 nm	0.076	0.012 (54)	0.066 (34)	0.393 (12)
	680–690 nm	0.226	0.035 (32)	0.133 (41)	0.603 (27)
	710–740 nm	1.258	1.258 (100)	-	-
**Desiccated**					
RT	560–640 nm	0.055	0.012 (75)	0.067 (19)	0.550 (6)
	680–690 nm	0.135	0.015 (44)	0.095 (33)	0.417 (23)
	710–740 nm	0.064	0.006 (52)	0.048 (35)	0.333 (13)
77 K	560–640 nm	0.073	0.015 (74)	0.084 (19)	0.689 (7)
	680–690 nm	0.137	0.022 (36)	0.105 (44)	0.407 (20)
	710–740 nm	0.193	0.015 (51)	0.084 (27)	0.748 (22)
16 K	560–640 nm	0.065	0.014 (74)	0.078 (20)	0.633 (6)
	680–690 nm	0.140	0.020 (35)	0.097 (41)	0.395 (24)
	710–740 nm	0.158	0.014 (57)	0.088 (26)	0.740 (17)
**Desiccated after RT→16 K→RT cycle**					
RT	680–690 nm	0.157	0.021 (43)	0.106 (26)	0.389 (31)
	710–740 nm	0.099	0.025 (69)	0.139 (20)	0.502 (11)
**Rehydrated desiccated after RT→16 K→RT cycle**					
RT	680–690 nm	0.150	0.037 (36)	0.130 (53)	0.620 (11)
	710–740 nm	0.100	0.016 (35)	0.077 (49)	0.364 (16)

Spectral decompositions of these data into a “blue” and a “red” component were performed after removing the orange band contribution (Fig. 2A and *Supplementary Material*, Fig. S6). At RT, the blue component is similar to that attributed to PSII in FL spectra of plants and green algae, located at 685 nm with a weak shoulder around 730 nm (Wientjes et al. 2017). At low temperatures, it remains largely unchanged, except a slightly more pronounced peak–shaped shoulder at 740 nm, which possibly originates from the monomeric PSII antenna complexes, as reported for *Arabidopsis thaliana* (Mascoli et al. 2020). The second component displays the characteristics of PSI emission spectrum (Lamb et al. 2018), comprising a band around 690 nm at RT, and an intense band at 720 nm accompanied by a shoulder near 690 nm at 77 K. It is of note that additional contributions from long–lived red–emitting states of the PSII antenna may contribute to this shoulder, and that the sharp band around 690 nm, present at 16 K, likely arises from such red–emitting states (Chmeliov et al. 2016). Upon lowering the temperature from RT to 16 K, the blue component decay slows down (Fig. 2B). The red component, faster at RT, experiences a prolonged growth followed by a very slow decay at 77 K and lower.

Overall, the RT results are consistent with a highly efficient photochemical excitation trapping in PSI and a typical physiological regime. At cryogenic temperatures, slower excitation transfer through the PSII antenna prolongs the decay kinetics, while long–lifetime fluorescence from red–emitting antenna states in both PSI and PSII appear within the given time window of 800 ps.

### Dehydrated moss

As reported before, dehydrated *S. caninervis* moss displays extremely weak steady-state fluorescence ((Li et al. 2010); *Supplementary Material*, Fig. S1). At RT, its time-resolved FL spectrum exhibits a broad emission with a maximum at 685 nm, extending towards the red spectral range (Fig. 1A and *Supplementary Material*, Fig. S2), which indicates a stronger contribution from PSI. The emission in the orange region appears more intense, revealing the important overall decrease of the FL intensity in the whole 680–720 nm region. At cryogenic temperatures, an intense new band appears at 718 nm, slightly blue–shifted and significantly more intense compared to corresponding band in the hydrated sample.

The FL decay kinetics reveal larger temperature variations in FL decay lifetimes than in the hydrated sample (Fig. 1B, middle). At 77 K and below, FL in the 710–740 nm region decays up to one order of magnitude slower than in the 680–690 nm region (see Fig. 1C; Table 1), and overall, both spectral regions exhibit faster decay kinetics at all temperatures.

At RT, the FL kinetics in both spectral regions are best described by a biexponential decay function, with a dominant fast decay lifetime (~ 60 ps), which remains unchanged, and a slower decay lifetime, becoming 20% faster in both spectral regions as compared to the hydrated sample (Table 1). At 77 K, the 680–690 nm region exhibits three–exponential decay, with two decay lifetimes approximately 40% faster than observed in the hydrated sample, and an additional very fast component of ~ 30 ps. In the 710–740 nm region, an additional decay component of 170 ps also appears, while the slow lifetime remains unchanged as compared to the hydrated sample. At 16 K, the decay becomes mono–exponential in this region and is approximately one–third faster than in hydrated moss.

Spectral decomposition shows that at RT, both components in the dehydrated sample decay similarly (Fig. 2C). Compared to the hydrated sample, the PSI–dominated red component exhibits only slightly shorter FL lifetime, whereas the PSII–dominated blue component decays significantly faster. At 77 K and below, the decay of the blue component remains largely unchanged (becoming only slightly slower at 16 K), whereas the red component shows an initial growth followed by a slow decay, however, faster than observed in the hydrated sample. Overall, dehydration thus induces a significant fluorescence quenching in all spectral regions in *S. caninervis*.

### Desiccated moss and rehydration

Upon desiccation, the steady-state fluorescence drops even more (*Supplementary Material*, Fig. S1). At RT, the 800-ps integrated FL spectrum in the red region closely resembles that of the hydrated sample with a peak at 685 nm, clearly distinct from the dehydrated one (see Fig. 1 and *Supplementary Material*, Fig. S2). Its overall FL intensity drops as compared to the dehydrated sample, as indicated by the apparent increase of the fluorescence in the orange region. Upon cooling at 77 K, a weak, additional band at 718 nm appears, located at the same spectral position as in the dehydrated sample, and both spectral regions exhibit rapid decay at earliest time independently of temperature below 77 K (Fig. 1B).

All FL decays are three–exponential (Table 1), indicating a somewhat higher degree of inhomogeneity in this sample compared with hydrated moss, for which a two-exponential fit was sufficient. The FL lifetimes in the 680–690 nm region are temperature–independent, with decay components of approximately 20, 100, and 400 ps at all measured temperatures. In the 710–740 nm range, the kinetics below 77 K becomes significantly slower than at RT, with decay lifetimes of approximately 15, 85, and 740 ps—all significantly faster than in hydrated or dehydrated moss.

Upon spectral decomposition, the PSII–dominated blue component appears temperature independent (Fig. 2D). At RT, it decays more slowly than the red component—a behaviour similar to what observed in the hydrated sample. In contrast, the red component decays much more slowly at low temperatures than at RT. It should be noted that a very fast decay signal at the beginning of the red–component kinetics is most likely a mathematical artefact of the decomposition procedure, though it can also to some extent reflect the ~ 15 ps lifetime component (cf. Table 1). Overall, in the desiccated sample, the blue component is slightly more quenched than in the hydrated sample, but less than in the dehydrated one. At RT the red component decays faster with increasing dehydration, but below 77 K, its behaviour is similar as in the dehydrated situation.

This sample was then rehydrated after cooling down to 16 K and further investigated (see *Supplementary Material*, Fig. S3). The time–integrated FL spectrum of such rehydrated sample at RT is shown in Fig. 1A (left; gray line). It exhibits nearly identical FL spectra to that of the hydrated sample, with, however, a slightly faster decay in both spectral regions. The initial rise in the FL kinetics also appears to be largely restored in the rehydrated sample (Fig. 1D; gray lines). Although the recovery is not complete, these results indicate that the desiccated sample withstands cooling to 16 K and subsequent rehydration, as its FL properties are largely restored.

## Discussion and conclusions

### Dehydration and *S. caninervis* fluorescence kinetics

Progressive dehydration of *S. caninervis* induces a dramatic decrease of its steady-state fluorescence intensity ((Li et al. 2014), *Supplementary Material*, Fig. S1). This may indicate that the chlorophylls associated with PSII and/or PSI become efficiently quenched upon dehydration. We performed time-resolved FL measurements in the sub-nanosecond time window, which yield information on every event occurring with kinetics longer than ~ 10 ps. Such experiments provide a direct view of the early excitation dynamics in *S. caninervis*, and, actually reveal emission from both PSII and PSI in dried moss, which are largely invisible in the steady-state FL spectra. This enables us to quantify quenching processes that remain hidden in the steady-state measurements. In hydrated moss at RT, the biexponential FL kinetics in all regions and their relative contributions are consistent with lifetimes reported for low–light grown *Arabidopsis thaliana*, where plants possess extra LHCIIs and exhibit PSII lifetimes of ~ 310 ps (Wientjes et al. 2013) and PSI-LHCI complexes show average lifetimes of 60 ps (Bos et al. 2023; Wientjes et al. 2011). In dehydrated moss, the FL kinetics remain biexponential, but the fast component (~ 60 ps) increases dramatically in amplitude, accounting now for ~ 94% of the signal, while the lifetime of the slower component decreases to ~ 250 ps and contributes only to ~ 6% of the observed signal. As a result, the mean excitation lifetime decreases more than two–fold upon dehydration, indicating a strong fluorescence quenching. However, this quenching alone cannot account for the ten– to twenty–fold drop in steady-state FL intensity. Further drying of the moss results in an additional drop in steady-state FL, but results in a partial uplift of the quenching in the PSII region alongside enhanced quenching of PSI. In such desiccated sample, which is highly heterogeneous and yields weak time-resolved FL signals, the FL decay kinetics observed cannot either account for the additional intensity drop observed in its steady-state fluorescence.

Below 77 K, photochemistry in photosynthetic organisms is effectively blocked, and, as a result, excitations remain confined in the antenna. Such low temperature measurements may thus help to further understand how dehydration affects the organization and energy transfer pathways of the photosynthetic apparatus. In the dehydrated state, the 680–690 nm FL kinetics remain strongly quenched regardless of temperature (Fig. 1C), indicating that the dehydration–induced quenching persists even when photochemistry is blocked at very low temperatures. Desiccation causes as well additional quenching in the 710–740 nm region at cryogenic temperatures (Fig. 1C). To better analyze these decay behaviors, we performed a simultaneous spectral decomposition of the time-resolved FL datasets across all hydration states by decomposing the data into two dominant spectral components with hydration–independent lineshapes. The blue component corresponds to the typical PSII emission at 685 nm and the red component mainly corresponds to PSI (Fig. 2A), however, at low temperatures, accompanied by far–red–emitting states arising from the PSII antenna that act as long–lived radiative traps (Chmeliov et al. 2016; Mascoli et al. 2020). At low temperature, a pronounced rise phase in the red component is observed for both the hydrated and dehydrated samples. This delayed fluorescence indicates that PSII cannot trap the excitation energy anymore when photochemistry is blocked, as charge separation is inhibited, and that instead, part of the excitation migrates toward the red–emitting antenna species of PSII and/or to PSI. This suggests a degree of connectivity between the two photosystems, which must consequently share part of the photosynthetic antenna network. This connectivity would also explain the obtained longer PSI–associated FL decay kinetics (compared to the typical lifetimes reported for plants (Akhtar et al. 2025; Liu et al. 2025), that were previously observed in the model moss *Physcomitrium patens* (Verhoeven et al. 2026).

### Dehydration and *S. caninervis* photosynthesis

Our results demonstrate that hydrated *S. caninervis* shows classical photosynthetic energy transfer behavior at all temperatures. In the dehydrated state, the fluorescence observed possesses a significantly lower intensity and profoundly altered excitation dynamics. A fraction of both PSI and PSII remain present—both display fluorescence at every temperature. The dominant decay component in both PSII and PSI emission regions becomes as short as 60 ps, indicating that nearly all excitations are rapidly trapped by highly efficient quenchers. When water becomes limited, charge separation in PSII slows down, causing excess excitation energy to accumulate even under low–light conditions. As this excess energy promotes the formation of reactive oxygen species, it must rapidly be dissipated through non-photochemical pathways to avoid photodamage. It is well–established that, in the presence of high–light or abiotic stress, PSII fluorescence is rapidly quenched by the so–called Non-Photochemical Quenching (NPQ) (Ruban 2016). The latter comprises several phases, the most studied being the fast–reversible one, termed energy–dependent quenching (qE), which was modelled by LHCII aggregation (Shukla et al. 2020). However, LHCII–aggregation quenching is released at very low temperature (~ 77 K) (Zubik et al. 2013), while it is not the case for dehydrated *S. caninervis* (Fig. 2C; Table 1). This indicates that a different mechanism of energy quenching occurs in this organism. Also, in dehydrated *S. caninervis*, the longer–lifetime component of PSI fluorescence is reduced at RT, and additional fast decay component appears below 77 K (compared to hydrated sample), indicating quenching which is not released at very low temperatures. Although PSI-associated quenching at 77 K has been reported previously in winter-acclimated *Taxus cuspidate* (Yokono et al. 2008), the exceptionally strong shortening of the red-component lifetime observed in desiccated *S. caninervis* suggests a distinct and more efficient photoprotective mechanism associated with severe dehydration. Such PSI-associated quenching of this magnitude under desiccation, to our knowledge, has not yet been reported. Possible mechanisms for such process might involve conformational changes in the LHCs, or energy spillover. The fact that this quenching persists when photochemistry is frozen suggests a phenomenon of static structural quenching occurring in the antennae.

Further desiccation of *S. caninervis* induces the appearance of additional lifetime components reflecting a high inhomogeneity in the sample, and the weakness of the signals measured reflects the small amounts of photosystems-associated pigment–protein complexes that remain capable of absorbing light and emitting fluorescence. We do not see any clear spectral blueshift of the main peak that would indicate the presence of free Chl. The PSII fluorescence partially recovers the lifetime they display in hydrated conditions (Fig. 2D) but the PSI emission remains strongly suppressed, suggesting the formation of structurally stabilized quenching sites that maintain photoprotection during prolonged desiccation. Notably, PSI are extremely quenched in these conditions, displaying an average lifetime of 193 ps at 77 K, i.e. 85% faster than for the hydrated moss at this temperature. Such desiccation-induced quenching involving both PSI and PSII contrasts with the response observed in the lichen *Parmelia sulcata*, where quenching under severe dehydration primarily targets PSII, while PSI kinetics remain largely unaffected (Veerman et al. 2007).

Importantly, the changes in the fluorescence dynamics observed upon dehydration/desiccation, does not fully account for the decrease observed in steady-state FL. Dehydration induces a 10-to-20-fold decrease of the steady-state FL intensity, while our time-resolved experiments may account for a 4-to-6-fold effect only. Additionally, desiccation induces a further decrease of the steady-state FL of *S. caninervis*, which cannot be explained either by the changes in fluorescence dynamics. The mismatch between lifetime shortening and intensity loss suggests that the presence of additional mechanisms at stake in the *S. caninervis* photosynthetic membrane upon dehydration/desiccation. Decrease in steady-state FL can also originate from a loss in absorption. Dehydration induces a visible colour change from green to dark brown (Fig. 1A) of *S. caninervis*, suggesting major alterations in the pigment organization within the chloroplast. During dehydration, chloroplasts shrink and migrate toward the cell centre, while their thylakoid membranes become more irregularly arranged (Li et al. 2014; Wu et al. 2012). These structural rearrangements may alter the optical properties of moss by reducing light penetration deeper into thylakoid layers, decreasing the number of chlorophylls excited under identical illumination conditions, and consequently the intensity of the emitted fluorescence. The formation of yet–unidentified, highly quenched chlorophyll aggregates, potentially with reduced absorption, could as well account for the observed drop in FL upon dehydration. However, this would imply that, in the desiccation state, nearly all chlorophyll molecules in the thylakoid membrane would dissociate from their host proteins to form such aggregates. It further implies that these pigments must be rapidly reassembled into active complexes within the first few minutes after rehydration, which seems unlikely on such a short timescale. It should be noted that, in none of the samples measured here, FL kinetics were consistent with free chlorophyll molecules observed. Therefore, these proposed quenched aggregates would need to incorporate essentially 100% of the chlorophyll released from protein binding sites, which is inconsistent with all known mechanisms of spontaneous chlorophyll aggregation, as such processes typically result in equilibria among multiple association states.

Another possible explanation for this mismatch is the appearance, upon dehydration, of extremely efficient quenching sites (“superquenchers”) that deactivate excitations on timescales faster than those resolved in our experiment (~ 10 ps). In isolated high light–inducible proteins D (HLiDs), members of the LHC family, specialized in photoprotection and characterized by strong quenching, trapping of chlorophyll excited states by carotenoids has been reported to occur with kinetics as fast as 2.1 ps (Staleva et al. 2015). Such a mechanism could explain the apparent decrease in steady-state FL, however, even in the presence of large amounts of such quenching proteins in the photosynthetic membranes, excitation energy would still require to migrate from the LHC to these proteins, which should produce a detectable FL signal on the picosecond timescale. Experiments on LHCII isolated from maize under hydrostatic pressure (van Oort et al. 2007) have shown that applying moderate pressure induces a decrease in fluorescence proportional to the applied pressure. This suggests that external physical parameters may induce the formation of quenching sites within the LHCII structure. Therefore, dehydration may induce a general quenching effect across LHCs present in the photosynthetic membranes of *S. caninervis*. Such a situation, combining ultrafast quenchers with short excitation migration times to the quenching sites, could account for both the observed decrease in steady-state FL and the time-resolved data presented here. Testing the presence of such ultrafast quenchers in dehydrated *S. caninervis* requires higher–resolution time-resolved FL measurements such as FL up-conversion.

Although the molecular origins of the different quenching mechanisms occurring upon dehydration in *S. caninervis* remain unclear, our data suggest a hierarchical scheme describing how photoprotection is acquired or relieved during dehydration and rehydration in this moss. In a highly dehydrated state, either the optical properties of the photosynthetic membranes are affected, or a general ultrafast quenching mechanism is established in this membrane. At intermediate dehydration, this quenching is partially relieved, and activity of both photosystems becomes detectable. However, in this metabolic state, additional quenching mechanisms appear that actively protect photosystems II and I; which are fully relieved only upon additional rehydration. From a photoprotection perspective, the transition of *S. caninervis* from a highly dehydrated to a fully hydrated state thus involves progressively milder photoprotective mechanisms, accompanying this transition while still preventing damage to the photosystems.

## Supplementary Information

Below is the link to the electronic supplementary material.

Supplementary Material 1

## Data Availability

The data presented in this manuscript will be provided by the corresponding authors upon request.
