# Supplementary material for: Photoprotection in a desert moss: dynamic excitation quenching during the hydration cycle of the Syntrichia caninervis
Source: Photosynth Res. 2026 Jul 10;164(4):39. doi: 10.1007/s11120-026-01230-4 (PMC13354704; doi:10.1007/s11120-026-01230-4)
Supplement: Supplementary file 1 — Supplementary Material 1 [file 11120_2026_1230_MOESM1_ESM.pdf]

Supplementary Material

Photoprotection in a desert moss: dynamic excitation quenching during the hydration cycle of the *Syntrichia caninervis*

Simona Streckaite[1*], Jevgenij Chmeliov[1,2], Vilius Čirgelis[1,2], Marius Franckevičius[1], Lena Golubewa[1,2], Benfeng Yin[3], Danielis Rutkauskas[1], Chunhong Yang[4], Leonas Valkunas[1], Yuanming Zhang[3*], Bruno Robert[5*]

[1]*Department of Molecular Compound Physics, Center for Physical Sciences and Technology, Saulėtekio Ave. 3, Vilnius, 10257, Lithuania*
[2]*Institute of Chemical Physics, Faculty of Physics, Vilnius University, Saulėtekio Ave. 9, Vilnius, 10222, Lithuania*
[3]*State Key Laboratory of Ecological Safety and Sustainable Development in Arid Lands, Xinjiang Institute of Ecology and Geography, Chinese Academy of Sciences, Urumqi, Xinjiang, 830011, China*
[4]*Key Laboratory of Vegetation and Environmental Change, Institute of Botany, Chinese Academy of Sciences, Beijing, 100093, China*
[5]*Institute for Integrative Biology of the Cell, Université Paris-Saclay, CEA, CNRS, Gif-sur-Yvette, 91198, France*

Additional fluorescence and spectral decomposition data:

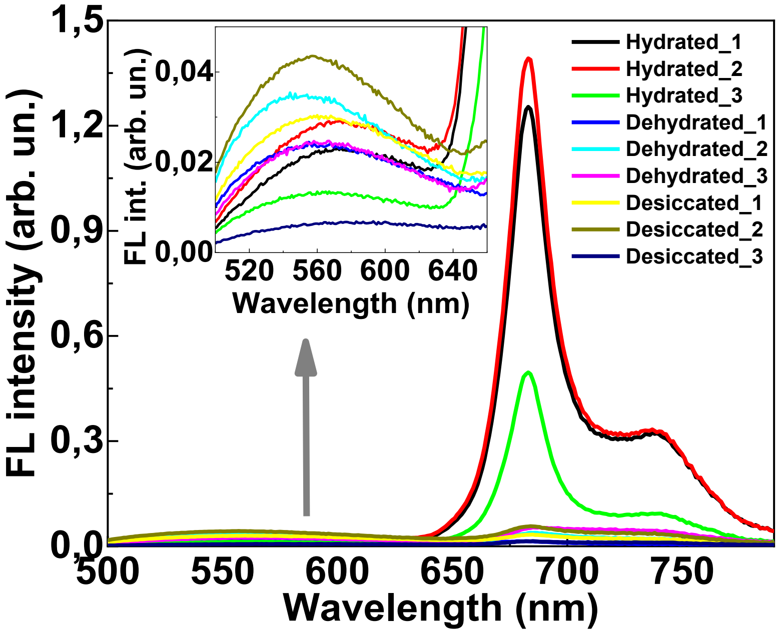

Fig. S1 Steady-state FL spectra of hydrated, dehydrated and desiccated *S. caninervis*, measured at room temperature. Inset shows enlarged orange region; $\lambda_{exc} = 470$ nm

* simona.streckaite@ftmc.lt; zhangym@ms.xjb.ac.cn; Bruno.ROBERT@cea.fr

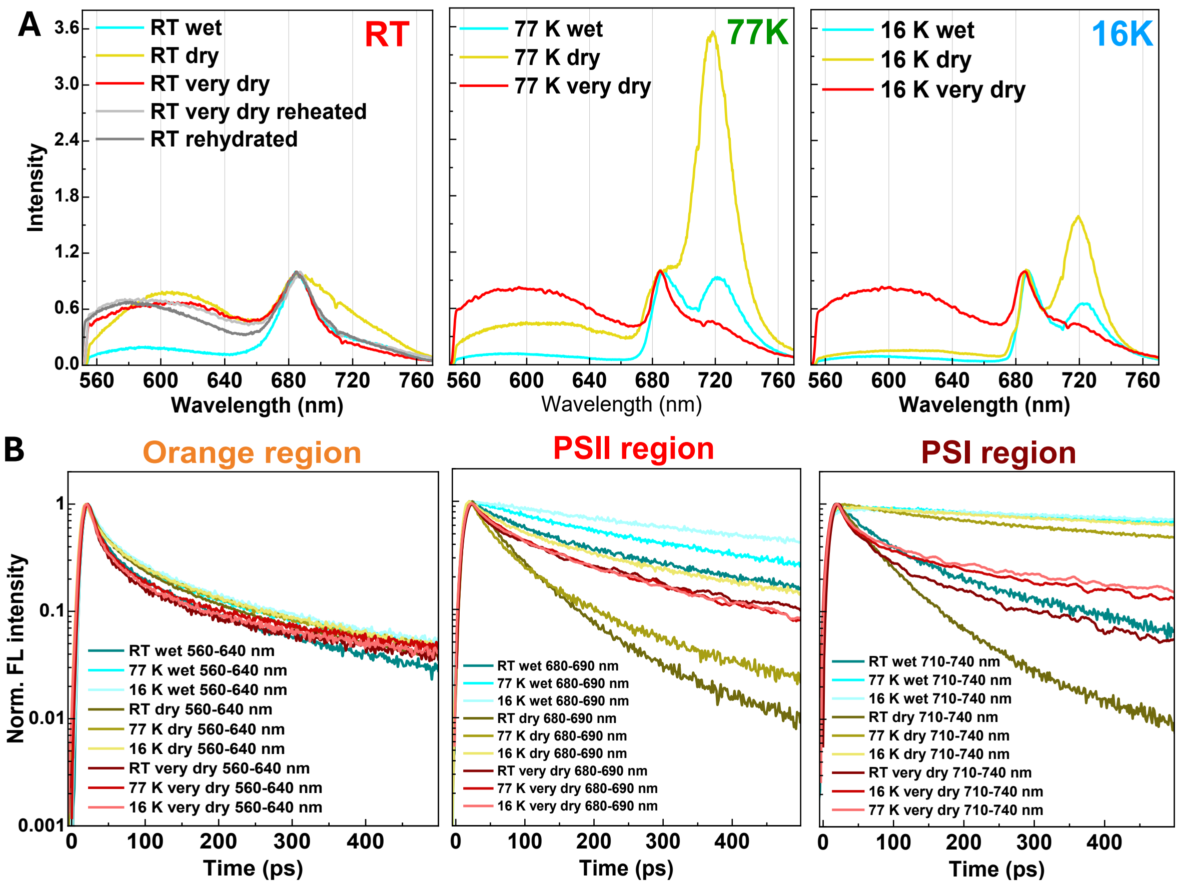

Fig. S2 800-ps integrated FL spectra, normalized to the 685 nm band (**A**), and normalized FL kinetics of 560–640 nm, 680–690 nm and 710–740 nm regions (**B**) for hydrated (wet), dehydrated (dry) and desiccated (very dry) *S. Caninervis* at RT, 77 K and 16 K. Measurement window—800 ps, laser repetition rate—80 MHz; $\lambda_{exc} = 515$ nm

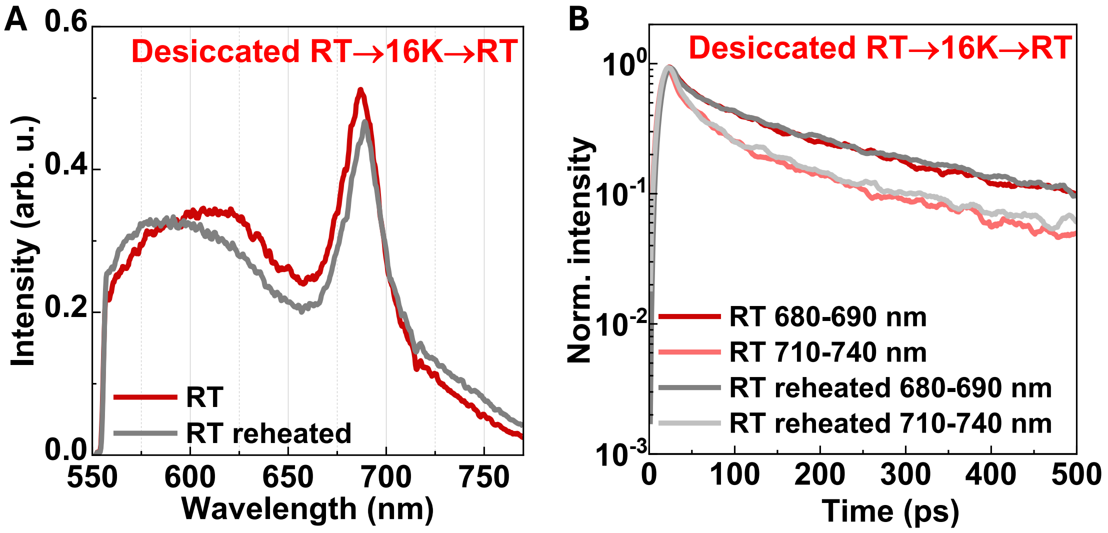

Fig. S3 800-ps integrated FL spectra (**A**), and normalized FL decay kinetics (**B**) of 680–690 nm and 710–740 nm regions of desiccated *S. Caninervis* at RT before cooling it down to 16 K (red lines) and after reheating it back to RT (gray lines). Measurement window—800 ps; laser repetition rate—80 MHz; $\lambda_{exc} = 515$ nm. All spectra are normalized to the measurement time

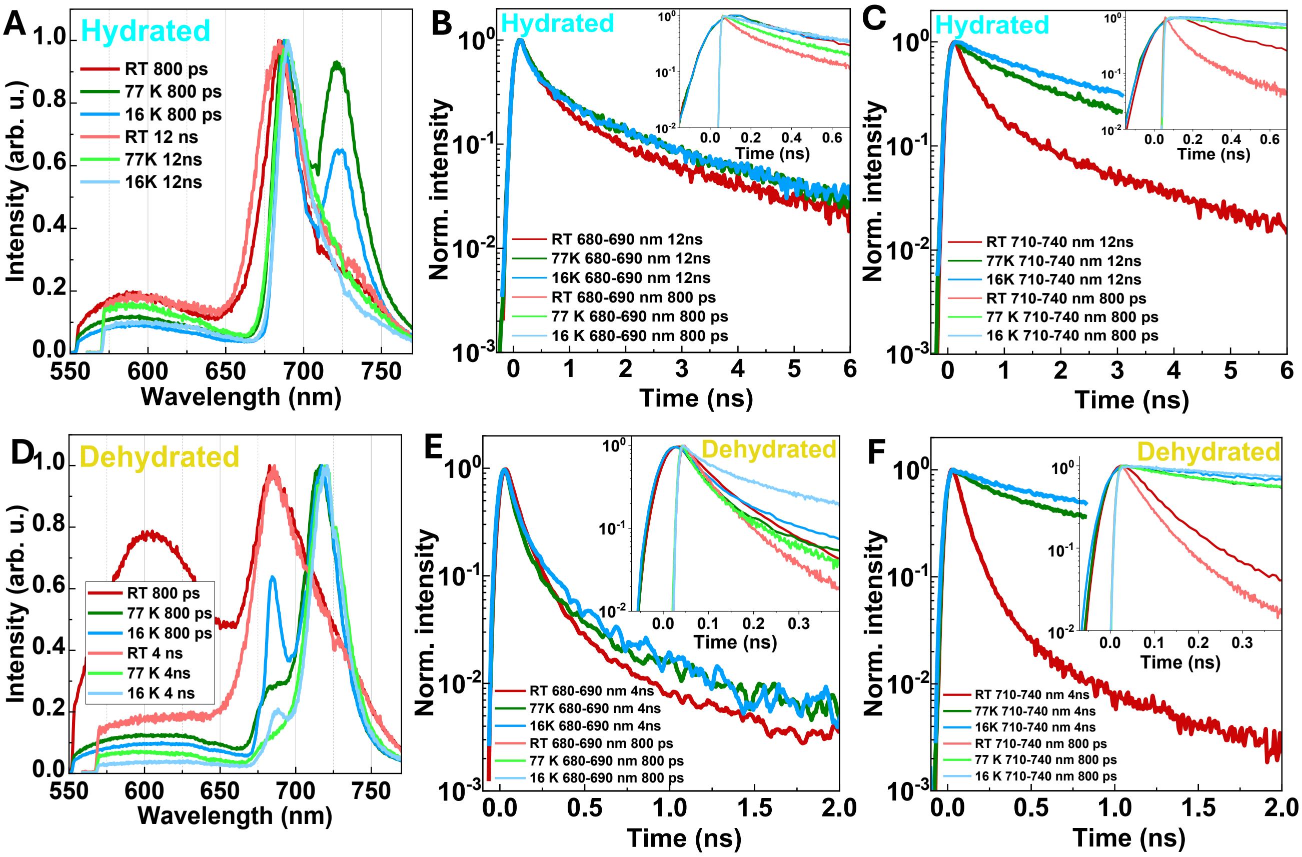

Fig. S4 Comparison of 800 ps, 4 ns and 12 ns time-resolved FL measurements of dehydrated and hydrated *S. Caninervis* at different temperatures. **A**, **D**: normalized integrated FL spectra; **B, C, E, F**: normalized FL decay of 680-690 nm and 710-740 nm regions at RT, 77 K and 16 K. Insets show initial 400 and 700 ps of FL kinetics for dehydrated and hydrated samples, respectively. $\lambda_{exc} = 515$ nm

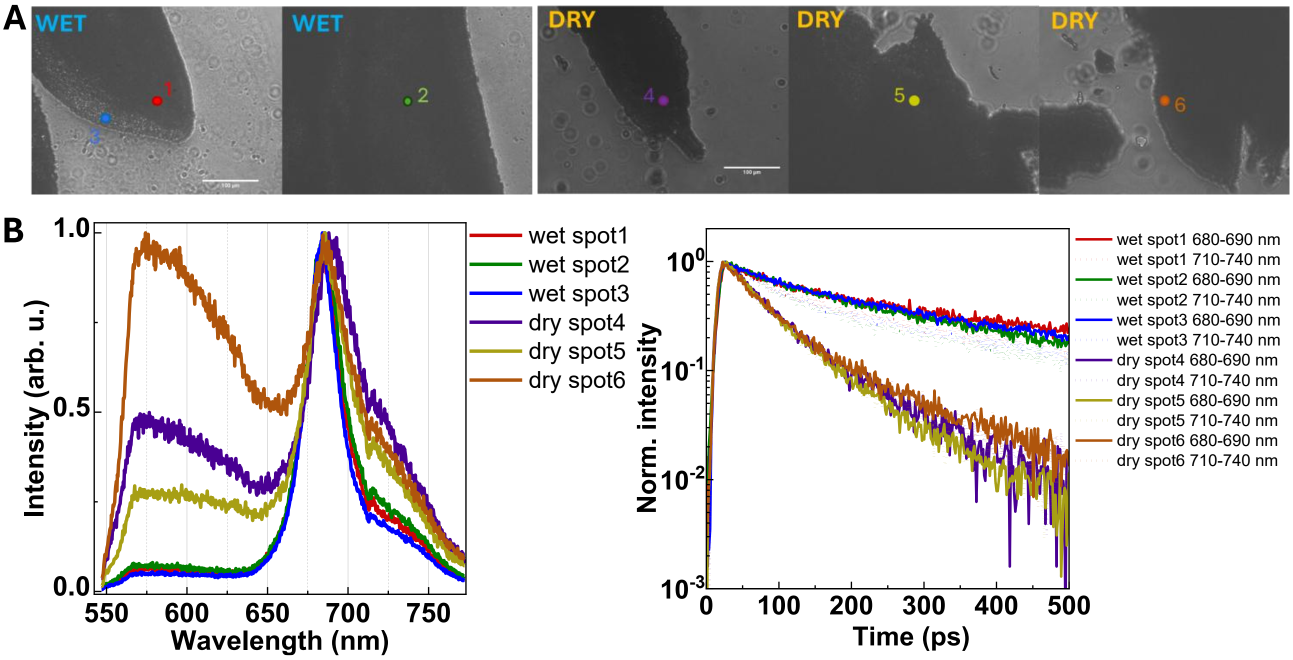

Fig. S5 Comparison of RT fluorescence properties of different spots of hydrated (wet) and dehydrated (dry) *S. Caninervis*: **A**: Wide field microscopy images at 10× magnification. **B:** 800-ps integrated FL spectra (left), and normalized FL decay kinetics (right) of 680–690 nm and 710–740 nm regions of the corresponding spots in **A**. Measurement window—800 ps; laser repetition rate—80 MHz; $\lambda_{exc} = 515$ nm. All spectra are normalized to the measurement time

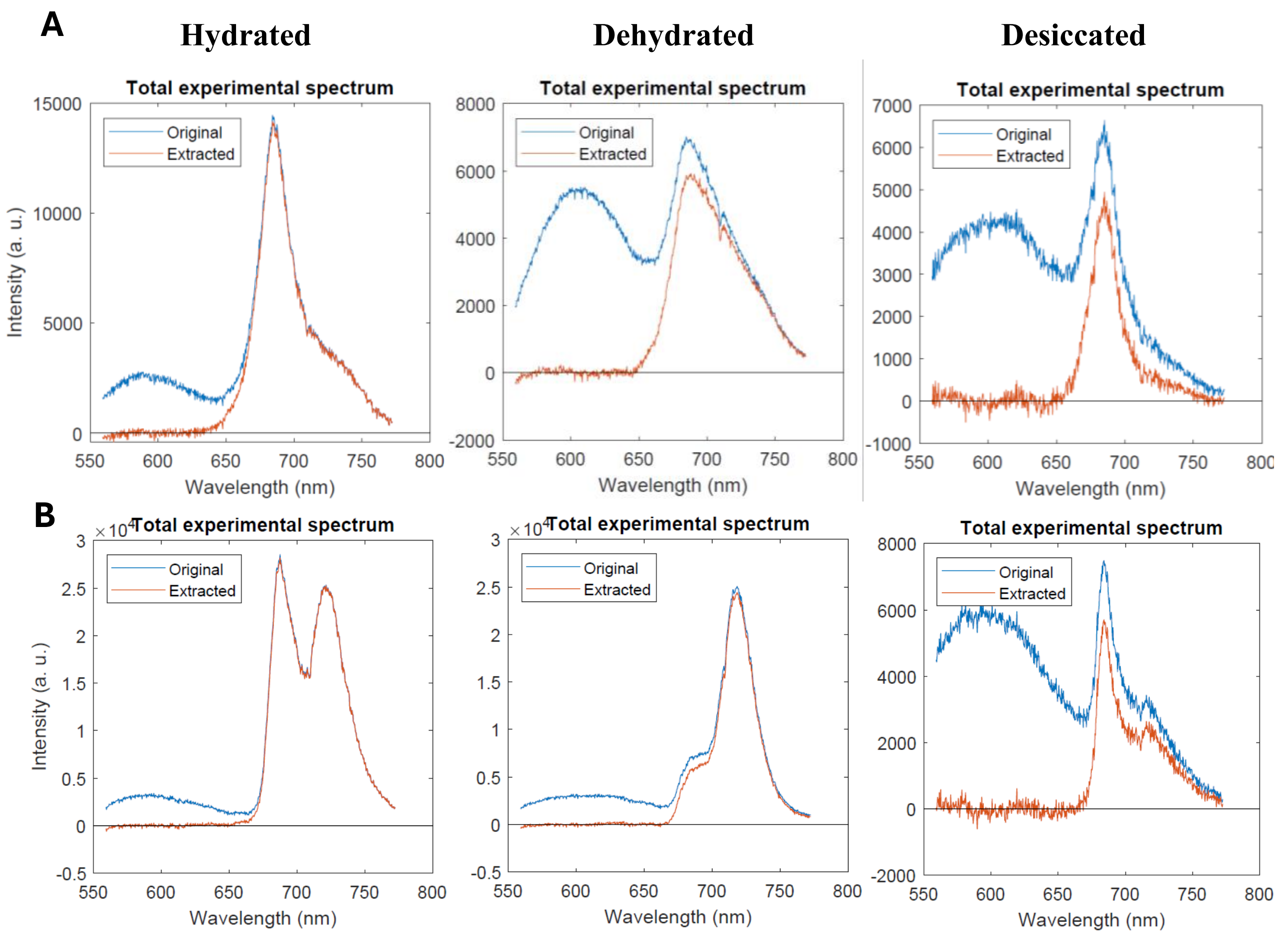

Fig. S6 Total time-integrated experimental FL spectrum used for spectral decompositions after the removal of orange region for hydrated, dehydrated and desiccated *S. caninervis* measured at RT, 77 K and 16 K

Table S1 FL lifetimes with their amplitudes in brackets of each component of multiexponential decay fits of FL kinetics of spectral decomposition components and regions of interest of hydrated, dehydrated and desiccated *S. caninervis* measured at different temperatures and at different time windows. $\lambda_{exc} = 515$ nm

	**Component**	**τ_1 [ns] (A_1 [%])**	**τ_2 [ns] (A_2 [%])**	**τ_3 [ns] (A_3 [%])**
**Spectral decomposition kinetics of 800 ps time window measurements**				
**Hydrated**				
RT	Blue	0.108 (58)	0.402 (42)	-
	Red	0.057 (81)	0.335 (19)	-
77 K	Blue	0.132 (44)	0.608 (56)	-
	Red	0.037 (-68)*	2.115 (168)	-
16 K	Blue	0.189 (38)	0.765 (62)	-
	Red	0.079 (-80)*	2.589 (180)	-
**Dehydrated**				
RT	Blue	0.063 (100)	-	-
	Red	0.057 (96)	0.282 (4)	-
77 K	Blue	0.049 (100)	-	-
	Red	0.016 (-324)*	0.822 (424)	-
16 K	Blue	0.077 (100)	-	-
	Red	0.044 (-114)*	1.280 (214)	-
**Desiccated**				
RT	Blue	0.134 (49)	0.379 (51)	-
	Red	0.070 (96)	0.917 (4)	-
77 K	Blue	0.150 (90)	0.808 (10)	-
	Red	0.003 (85)**	1.1113 (15)	-
16 K	Blue	0.139 (84)	0.521 (16)	-
	Red	0.005 (83)**	0.906 (17)	-

12 ns time window measured kinetics				
Hydrated				
RT	680-690 nm	0.237 (3)	0.889 (46)	3.562 (51)
	710-740 nm	0.223 (3)	0.877 (46)	3.352 (51)
**4 ns time window measured kinetics**				
**Dehydrated**				
RT	680-690 nm	0.064 (88)	0.211 (11)	1.556 (1)
	710-740 nm	0.051 (78)	0.139 (21)	0.958 (1)

*Negative amplitudes describe initial rise of the component ("delayed fluorescence")

**Very fast decay signal at the beginning of the red–component kinetics is most likely a mathematical artefact of the decomposition procedure

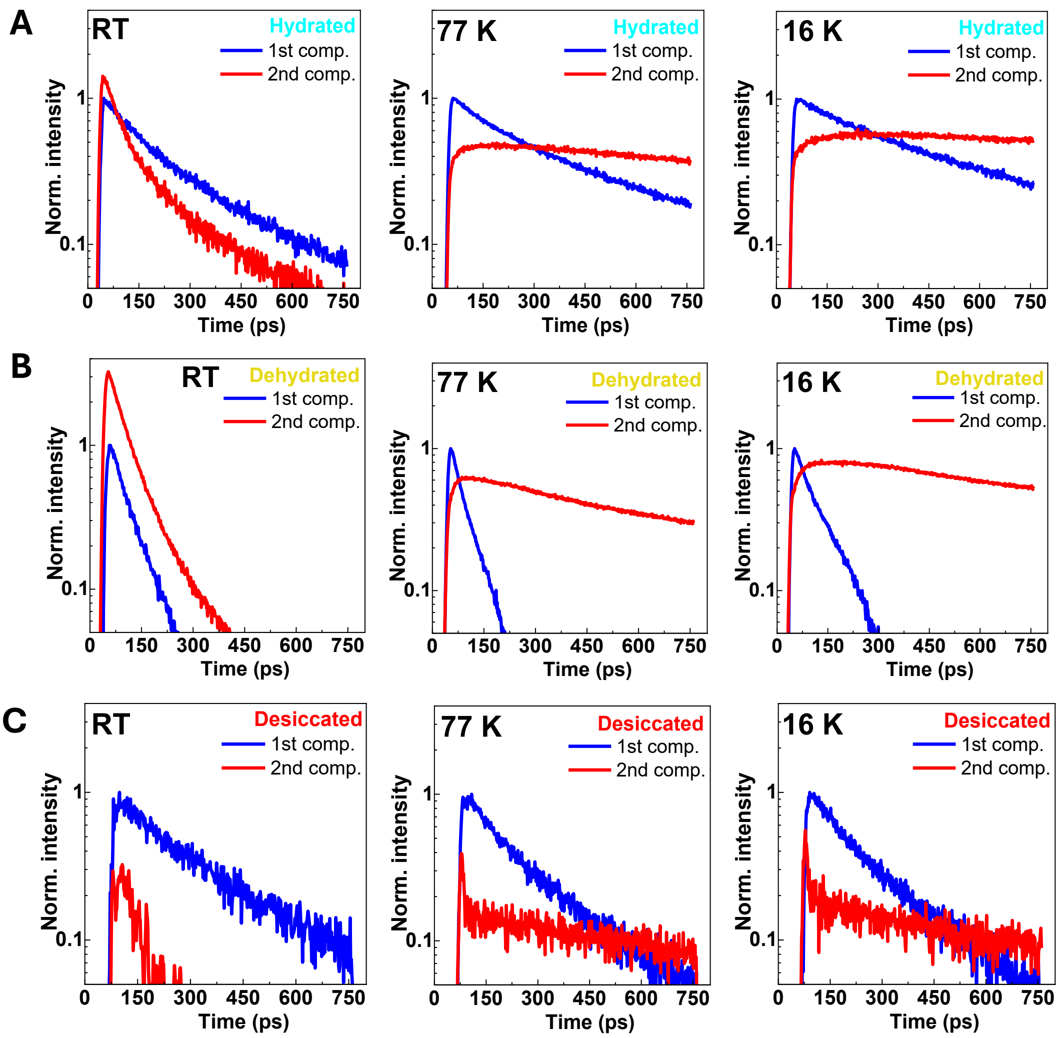

Fig. S7 FL kinetics corresponding to the components of spectral decompositions of hydrated, dehydrated and desiccated *S. caninervis* samples at RT, 77 K and 16 K. The orange region was excluded from all spectral decompositions
